# Crime, punishment, and detention in secure psychiatric hospital

**DOI:** 10.1177/00258024241256423

**Published:** 2024-05-22

**Authors:** Andrew Shepherd

**Affiliations:** 1 5292 Division of Psychology and Mental Health, University of Manchester, Manchester, UK; 2 Secure Care Services, Guild Lodge Secure Hospital, Preston, UK

**Keywords:** Expert witness, forensic psychiatry, legal system, law, human rights

## Abstract

Many legal jurisdictions offer some form of hospital diversion and disposal as an alternative to incarceration in prison for mentally disordered offenders. Such diversion is commonly understood as offering a non-punitive alternative in terms of sentencing decisions. However, complete loss of responsibility with respect to acts of violence is rare and indicative of extreme degrees of mental disorder. This raises challenges for sentencers when considering disposal options. From the perspective of the patient and healthcare providers while hospital may be framed as non-punitive, it still involves marked loss of freedom and rights. In this essay, it is argued that failure to acknowledge the punitive element, inherent in hospital detention, risks its repression, and a false dichotomy being established with prison being seen as solely punitive and hospital as solely therapeutic. It is suggested that this division is unhelpful, even potentially harmful, and that a synthesis as solution to this dialectic opposition may be generative in terms of therapeutic work in hospitals, clarification of the role of hospitals in terms of criminal justice disposal, and greater transparency in relation to multi-agency working and the social circumstances of patients detained in secure hospitals. Further work to understand this process is suggested with a particular emphasis being placed on the experience of specific groups of patients, such as women, who may find themselves in a notably precarious state within secure care.

*Our unconscious will murder even for trifles … it knows no other punishment for crime than death.* ^1^

Clinical experience suggests that, in the face of restrictions of an individual's personal freedom and deprivation of liberty arising from involuntary detention within a psychiatric hospital, it is not uncommon for mental health practitioners to face charges of their sadistic punishment of the individual. This may be particularly true in secure hospital settings, where measures such as seclusion, segregation, and confinement are used with the aim of supporting safety and public protection.^2^ While debates around the purpose and appropriateness of such detention abound, many people accept that an individual who, in the context of an acute mental health crisis, has acted with violence towards others ought to experience some form of restriction, though the perception of a suitable setting may vary.^3^ The immediate defence against the charge of punishment therefore appears simple: detention is deemed appropriate and proportionate and is supported through regular reviews, for example Mental Health Review Tribunals. However, to what degree is there a possible justification to the distressed cry of punishment by the detained individual? To what degree are mental health units, and particularly secure care settings, punitive in their nature?

In this essay, I want to address this unpalatable question which, I argue, links more broadly with discussions in relation to sentencing and responses to acts of criminal violence when committed, or carried out, by mentally disordered offenders. To consider these points, I first set out the common tests of guilt and responsibility that inform sentencing decisions and the role of prisons as illustrative of the penal element of any sentence that is meted out. I then compare this pathway with the journey of an individual sentenced to secure hospital. I argue that there are uncomfortable factors in these processes that sit ill at ease with principles of ethical healthcare. I suggest that these tensions are illustrative and potentially generative with respect to a wider discourse around the nature of mental healthcare delivery in the face of violence. Throughout this discussion I will refer to ‘mentally disordered offenders’ – this term is used in keeping with Crown Prosecution Service in England and Wales sentencing guidance and other documents; it is used cautiously however with an awareness of its stigmatising nature as well as its potential to diminish the experiences of trauma and despair that are almost ubiquitous in the wider prison population.

In setting out this argument I draw on a constructivist approach to social understanding – suggesting that institutions, such as hospitals and prisons, exist within a wider social milieu and discourse and that their function can only be understood in the context of this discourse, being constructed through the way language is deployed in relation to them. In this way, we can reach a plurality of ‘understandings’ with respect to the role of complex institutions. That is, while hospitals and prisons are undeniably ‘real’, in their physical concrete construction, they are also mental and social constructs that we can relate to in different ways – for example as both sites of healing and punishment. I also suggest that there is merit in considering the dialectic tension that emerges in the ideas of punishment (as in prison incarceration) and treatment (in hospital) and that a synthesis of these opposing understandings may be generative in our appreciation of the social role of both institutions.

## Crime and punishment

In England and Wales, and in many jurisdictions internationally, the concepts of *actus reus* and *mens rea* are employed in the definition of individual guilt. That is the individual *carried out* the act in question and *intended to do so*. Variations around this theme exist – for example in relation to crimes of specific intent – but the underlying structure of the two components is preserved. If an individual is convicted of a crime, then a disposal or sentence is awarded that will hold some form of punitive element. Prison sentences are perhaps the clearest illustration of this point – where an individual will receive a custodial tariff: however, the actual penal element of incarceration is not always immediately clear. Detention in prison is undeniably unpleasant, and particularly psychically toxic;^4^ however, in England and Wales, it is the loss of liberty and disenfranchisement [loss of citizenship and alienation from right to vote] for the length of the custodial sentence that forms the primary punishment itself with direct physical punishment no longer considered in this manner.^5^ Prisons, in fact, serve many roles in relation to criminal justice – which can, facetiously, be described in the ‘four (or five) Rs’: Retribution [penal element], restriction [public protection], rehabilitation [risk reduction], restoration [restorative justice], and redemption. The last of these is rarely stated as an aim of such organisations – but is too common a theme in the narratives of prisoners to ignore.^6^

For this argument, I want to highlight the complex and controversial social role occupied by prisons and point to their purpose beyond retribution: despite the understandable predominance of the penal element in the public imaginary.

## Mentally disordered offenders

Experience of mental distress and disorder can have wide reaching effects in relation to an individual's potential to carry out a crime, including acts of violence, and in their interaction with the criminal justice system. It bears repeating that, while mental disorder represents a risk factor for violence, those with experiences of disorder are more liable to be victims rather than perpetrators of violence. While an appreciation of an individual's mental health needs should inform each stage of the criminal justice process – from arrest, through Court, to sentencing and disposal – for the purpose of this argument I want to focus on the role of understandings of disorder in relation to the determination of guilt and sentencing decisions.

As a result of mental disorder an individual may be found not fit to plead – or stand trial – leading either to a delay in proceedings, while capacity is regained, or a trial of fact wherein the individual may be found to have carried out the act in question and then be disposed of appropriately, for example through a hospital or community order. The criteria with respect to fitness to plead, the Pritchard Criteria in England and Wales, are stringent and fitness is generally only lost in the face of significant degrees of psychopathology.

Most jurisdictions, internationally, hold some concept of ‘*not guilty by reason of insanity*’. For example, in England and Wales the, so-called, McNaughten principles apply^7^ – and similar statements can be seen internationally. In this context, insanity is a legal term rather than a medical one and is defined by:

1. Not knowing the nature of quality of the act carried out,
2. Not recognising that the act was ‘*wrong’* [clarified, in England and Wales R v Johnson (2007) as meaning ‘*legally’* rather than ‘*morally’* wrong].

Automatism may provide an alternative form of insanity defence. The presence of insanity does not necessarily preclude mens rea – as a person may still act in a recklessly criminal manner despite not necessarily recognising the nature of their acts. So defined, this represents a high threshold in terms of argument – since the degree of experienced psychopathology to extinguish understanding of the nature and legality of one's acts is significant. Once found not guilty by reason of insanity an individual must either receive a hospital order, community order, or be discharged from the charges that they face.

In cases of murder in England and Wales the defendant also has access to the partial defence of diminished responsibility wherein the presence of mental disorder is seen as abolishing the specific intent to kill or cause harm, leaving the defendant subject to the lesser charge of manslaughter.

### Sentencing decisions and mentally disordered offenders

In the absence of a full legal defence of insanity – or having been found not fit to plead and stand trial – a defendant may be found guilty of the alleged crime, either through plea or contested hearing: in such a situation, the sentencing official is charged with taking into account the individual's experience of mental disorder as one factor among many in relation to sentencing decisions. In England and Wales, the Mental Health Act (1983, revised 2007) affords the judge or magistrate options in terms of hospital disposal if they are in receipt of appropriate medical evidence. Generally, judges face a choice between community and hospital disposal – or so called ‘*hybrid orders’* [Section 45a Mental Health Act] which afford an initial detention in hospital as well as a defined sentence length during which time the individual is subject to return to prison if released from hospital.

When considering the potential options before them sentencing guidelines, such as those from the Sentencing Council for England and Wales,^8^ provide some factors for the sentencer to consider which, in essence, hinge on a recognition of the impact and stigma of mental ill health for the offender, as well as a gauge of *culpability*: The guidance highlights culpability as being a matter for the sentencer alone, though they may be aided in their deliberations by expert witness advice. The guidance provides recommendations with respect to a highly cognitive series of tasks that the sentencer may consider pertinent in addressing culpability including the defendant's ability:

1. To exercise appropriate judgement,
2. to make rational choices,
3. to understand the nature and consequences of their actions.

Deliberation in relation to these factors will be complex however – and it is apparent that understandings of complex experiences of trauma and mental disorder will be informed by a number of explicit and implicit factors and assumptions that, in essence, lead the sentencer to form a judgement with respect to the character and culpability of the accused. Factors such as intoxication with alcohol or illicit psychoactive substances at the time of the act in question may be seen as pertinent – but clearly sit in a complex relationship with broader concepts of mental disorder in terms of dependence, harmful use, and drug-induced psychotic states. Previous offending behaviour may also be taken as indicative – speaking to the character of the accused – but again can be seen as occupying a complex position in terms of assessing culpability for the current incident.

Personal experience in Courts, as well as a review of recent sentencing decisions, shows the great weight of deliberation undertaken by sentencers in relation to these decisions. For example, in sentencing remarks from a high-profile multiple homicide case, where the defendant was found guilty of manslaughter on the basis of diminished responsibility:

*I remind myself of the importance, where appropriate, of reflecting a penal element in the sentence but note, in passing, that the psychiatric evidence … is that you are unlikely ever to be released in any event.* [Sentencing remarks pp15-16 R v Calocane, case reference U20231322]

The call to the likelihood of the individual never being released is debatable here – since prognostication, with respect to likely recovery in the face of mental disorder, is complicated and the stress of a pending criminal trial renders any predictions during a period of remand even more fraught. This consideration of the penal element, along with considerations of public safety, suggests a dichotomy with hospital orders being seen as *non-punitive* versus the *punitive* prison sentence. Indeed, in taking account of this consideration, sentencers are reminded that the burden of imprisonment may fall more heavily on mentally disordered offenders such that a prison sentence may be seen as disproportionate. However, there is perhaps also an implication in this statement that the length of detention in hospital may take on the quality of a punitive act.

Reviewing the use of hybrid orders [Section 45a of the Mental Health Act], Peay^9^ highlighted factors that led to the decision to utilise a hybrid or standard mental health order versus a custodial prison sentence. She highlights the distinction, drawn by sentencing judges, between an offender as ‘*ill who required treatment: not a criminal who required punishment* [p29 ibid]. This, together with discussions of dangerousness and the requirement for monitoring post-sentence or after hospital discharge dominated discussions with respect to the role of Section 45a versus a conventional Section 37 [hospital treatment order]. Again, issues of culpability, responsibility, and retained culpability emerged – and are highlighted as of concern by Peay given their nebulous nature and the difficulty in determining degree of agency with respect to offending behaviour.

In summary then, in keeping with a likely broader lay understanding, sentencers when considering the role of hospital orders consider the degree to which the individual's experience of mental disorder informed their culpability at the time in question^10^: with a greater degree of disorder leading to a lessening of culpability and greater likelihood of decision to detain the individual in hospital. This decision is not simple and a range of factors appear to influence sentencing outcomes. The ultimate decision appears to rest on the dialectic distinction between hospitals as places of treatment versus prison as site of punishment. I argue that there is a tension here that will be explored further in the remaining sections of this essay.

### Secure hospitals as sites of recovery

Understandings of hospitals are generally constructed as sites of healing and recovery, not as sites of punishment. Recovery, in mental health practice, has come to take on a particular resonance emphasising themes of connection, hope, identity, meaning, and empowerment^11^ with reviews of forensic mental health settings emphasising the importance of an added theme of security.^12^ These concepts align appropriately with broad ethical principles – such as beneficence, non-maleficence, respect for autonomy, and justice – in healthcare practice.

However, immediately, the introduction of the theme of security, with implications for restriction, raises a challenge in relation to forensic mental health as the meaning of security can be seen in at least two different ways: pointing to the requirement for a place of safety for survivors of trauma and mental disorder^13^ as well as the needed restriction required for public safety in the face of violence emerging from experiences of mental disorder. This ethical tension is well recognised and generally not seen as being excessively in conflict with principles of healthcare delivery.^14^ Certainly, suggestions of hospitals as sites of punishment appear a long way from these understandings.

### Secure hospitals as sites of punishment

In terms of function, secure mental hospitals can be seen as sites of recovery with additional functions in terms of restriction [public protection in keeping with Mental Health Act frameworks], restoration [linking mentally disordered offenders with communities and taking steps towards rebuilding connection including the possibility of restorative justice when appropriate], and likely redemption. In this, their function is, in many ways, analogous to prisons – except putatively for the function of retribution.

However, it was argued above that – beyond the toxic nature of prison environments as sites of living, and secure hospitals are often themselves toxic in their own way – punishment was meted out in the form of lost liberty and disenfranchisement. Yet, liberty is also subject to deprivation within hospitals, and, in England and Wales, mentally disordered offenders are disenfranchised in terms of voting rights similarly to prisoners (patients detained under Part III, such as Section 37, of the Act have no right to vote in England and Wales).

In many ways, the pains of imprisonment, experienced by prisoners, particularly in terms of uncertainty with respect to future release, psychological assessment and development of personal autonomy,^15^ are also particularly pertinent to mental health patients in secure settings. Indeed, for many, the criteria for advancement within forensic mental health services, to progress towards eventual discharge, are seen as less transparent than the certainty that comes with a determinate prison sentence. Thus, mentally disordered offenders sentenced to hospital experience a loss of liberty, disenfranchisement from representation in democratic process, and a significant burden of imprisonment through uncertainty and the requirement to engage in painful identity work in coming to terms with oneself in line with the highly stigmatised identity of mentally disordered offender, alongside other identities such as substance user or trauma survivor.^16^ Is this not tantamount to punishment?

## Implications

This uncomfortable claim, that hospitalisation can be seen as a form of punishment, may be seen as an argument of sophistry – it could be claimed that what matters is the *intent* behind the deprivation of liberty: A benign intent in the case of hospitalisation versus the punitive intent of prison incarceration. Indeed, I would not seek to malign healthcare providers working in secure settings – the majority of whom do so with a clear goal of beneficence and justice. However, I do think that determining that motivation behind such detention may be more challenging – particularly in tragic cases such as in R v Calocane where the need for public protection weighs heavily in any consideration of justice in terms of outcome. Certainly, for the individual who is made subject to an extended deprivation of liberty, it may be difficult to interpret the State's intention behind their detention as benign. Clear acknowledgement of this punitive potential is important – its repression may contribute to the challenging dynamics that can emerge within secure hospital settings.

The argument presented here is intended as a provocation to illustrate the emergence of a potential unhelpful dialectic tension: hospitals viewed as entirely curative in their intent versus prisons being entirely punitive. This is clearly not a tenable position in the case of prisons – where the stated functions (rehabilitation, restriction, restoration) clearly extend beyond retribution. However, if we ignore the punitive aspect of restriction that comes with hospitalisation do we risk missing an important power dynamic and implication of our actions? If this is ignored, does it risk becoming a blind spot that leads to our missing the emergence of painful sadomasochistic responses within curative hospital settings? The challenges of working in such environments – and the risk of moving to a sadistic position – are clearly recognised.^17^ Acknowledgement of the violence inherent in depriving an individual of their liberty and clearly addressing this ambivalence may be beneficial and allow for a generative synthesis in terms of the role of both prisons and hospitals that – when clearly acknowledged – may benefit both in terms of social understanding and outcomes? Recognition of the importance of rehabilitation, beyond retribution, as a function of prisons is often highlighted. Perhaps there is something to be gained in clearly acknowledging the inherent violence attendant on depriving an individual of their liberty, and other rights, even when this is seen as being done out of benevolence. Clear articulation of this dynamic – through supervision, reflective practice, and in multi-agency working, may be beneficial in addressing the challenge of violence that is ubiquitous in forensic environments. For example, in relation to the use of seclusion within a secure hospital unit a multidisciplinary team member commented: ‘If *they don’t remain in seclusion how are they to know that they have done anything wrong?’*

If this argument were accepted then a review of the role of forensic hospitals would be called for – particularly in relation to admission criteria and acknowledgement of the challenges that appear, for example, in relation to those patients detained under civil sections (that is, those detained following Mental Health Act Assessment in England and Wales, not through criminal court). Questions may also be raised of the experience of patients not detained following conviction for an offence – but who have been admitted to secure settings because of incidents in other hospitals, a phenomenon that is perhaps particularly present for women in secure care settings.^18^ What are the implications of an unacknowledged punishment dynamic in such scenarios?

Further research and investigation – for example through ethnographic observation and decision-making studies – may be of benefit in betting exploring and mapping this tension which may also have implications in addressing the challenges attendant on multi-agency working within the criminal justice system through allowing a clear articulation of purpose of goals in the work as jumping off points for collaborative understanding and mutual support.

## References

[1] Freud S . Thoughts for the times on war and death, Trans Strachey, Standard Edition. London, England: Vintage Classics, 2001. Vol. XIV, p.297.

[2] Seppänen A Törmänen I Shaw C , et al. Modern forensic psychiatric hospital design: clinical, legal and structural aspects. Int J Ment Heal Syst 2018; 12: 58. 10.1186/s13033-018-0238-7 PMC6195744 30377440

[3] Niveau G Welle I . Forensic psychiatry, one subspecialty with two ethics? A systematic review. BMC Med Ethics 2018; 19: 25. 29636102 10.1186/s12910-018-0266-5 PMC5894191

[4] Shepherd A . Psychosocial meaning making in carceral spaces: a case study of prison and mental health care practice. J Psychosoc Stud 2021; 14: 139–151.

[5] Sykes G . The pains of imprisonment. In: The society of captives: a study of a maximum security prison. Princeton University Press, 1958.

[6] Maruna S . Making good: how ex-convicts reform and rebuild their lives. American Psychological Association, 2001.

[7] Montrose JL . The M’Naghten rules. Mod Law Rev 1954; 17: 383–386. http://www.jstor.org/stable/1091403

[8] Sentencing Council. https://www.sentencingcouncil.org.uk/overarching-guides/magistrates-court/item/sentencing-offenders-with-mental-disorders-developmental-disorders-or-neurological-impairments/#Section%20two:%20Assessing%20culpability (2020, accessed March 2024).

[9] Peay J . Sentencing mentally disordered offenders: conflicting objectives, perilous decisions and cognitive insights. SSRN Electron J 2015; 1.

[10] Mechanic D . Illness and social disability: some problems in analysis. Sociol Perspect 1959; 2: 37–41.

[11] Leamy M Bird V Boutillier CL , et al. Conceptual framework for personal recovery in mental health: systematic review and narrative synthesis. Brit J Psychiat 2011; 199: 445–452. 10.1192/bjp.bp.110.083733 22130746

[12] Shepherd A Doyle M Sanders C , et al. Personal recovery within forensic settings – systematic review and meta-synthesis of qualitative methods studies. Crim Behav Ment Heal 2016; 26: 59–75. 10.1002/cbm.1966 26095236

[13] Butler L Critelli F Rinfrette E . Trauma-informed care and mental health. Directions in Psychiatry 2011; 31: 197–210.

[14] Adshead G . Care or custody? Ethical dilemmas in forensic psychiatry. J Med Ethics 2000; 26: 302–304. 11055029 10.1136/jme.26.5.302 PMC1733273

[15] Crewe B . Depth, weight, tightness: revisiting the pains of imprisonment. Punishm Soc 2011; 13: 509–529.

[16] Hartwell S . Triple stigma: persons with mental illness and substance abuse problems in the criminal justice system. Crim Justice Policy Rev 2004; 15: 84–99.

[17] Adshead G . Psychiatric staff as attachment figures. Brit J Psychiat 1998; 172: 64–69. 10.1192/bjp.172.1.64 9534835

[18] Bartlett A Somers N Fiander M , et al. Pathways of care of women in secure hospitals: which women go where and why. Br J Psychiatry 2014; 205: 298–306. 25104832 10.1192/bjp.bp.113.137547

